# A new species of the genus *Truncocolumella* (Basidiomycota, Boletales) found in the Hengduan Mountains of China

**DOI:** 10.3897/BDJ.12.e128970

**Published:** 2024-11-21

**Authors:** Lin Li, Shanping Wan, Yun Wang, Naritsada Thongklang, Zonglong Luo, Shuhong Li

**Affiliations:** 1 College of Agriculture and Biological Science, Dali University, Dali, China College of Agriculture and Biological Science, Dali University Dali China; 2 School of Science, Mae Fah Luang University, Chiang Rai, Thailand School of Science, Mae Fah Luang University Chiang Rai Thailand; 3 Center of Excellence in Fungal Research, Mae Fah Luang University, Chiang Rai, Thailand Center of Excellence in Fungal Research, Mae Fah Luang University Chiang Rai Thailand; 4 College of Resources and Environment, Yunnan Agricultural University, Kunming, China College of Resources and Environment, Yunnan Agricultural University Kunming China; 5 New Zealand Institute for Crop and Food Research Limited, Mosgiel, New Zealand New Zealand Institute for Crop and Food Research Limited Mosgiel New Zealand; 6 Biotechnology and Germplasm Resources Institute, Yunnan Academy of Agricultural Sciences, Kunming, China Biotechnology and Germplasm Resources Institute, Yunnan Academy of Agricultural Sciences Kunming China

**Keywords:** false truffles, Hengduan Mountainous, ITS, morphological, taxonomy

## Abstract

**Background:**

During surveys of hypogeous fungi in the Hengduan Mountains, south-western China, three specimens of the genus *Truncocolumella* were discovered in Sichuan Province.

**New information:**

Morphological and molecular analyses revealed that the collections represent a new species, *Truncocolumellapseudocolumella*. This article describes the new species and discusses its relationship with the other two members of the genus.

## Introduction

*Truncocolumella* Zeller (Agaricomycetes, Basidiomycota) is a small genus of false truffles, established by Zeller in 1939 with the type species *T.citrina* Zeller (Zeller 1939). The main characteristics of this genus are: basidiomata depressed-spheroid to reniform, from a rhizomorphic base and with a central point of attachment; columella stump-like to dendroid, prominent; peridium persistent, not separating from the gleba; gleba pale white when immature, brown when mature; spores smooth, ellipsoid, as seen individually, almost hyaline. Another species, *Truncocolumellarubra*, was named in the same article and later recombined as *Gastroboletusruber* (Zeller) Cázares & Trappe (Cázares and Trappe 1991). The third species in this genus, *T.occidentalis* (Malençon) Malençon & Zeller, was reclassified from *Dodgeaoccidentalis* Malençon (Malençon and Zeller 1940). Subsequently, T.citrinavar.citrina Zeller and T.citrinavar.separabilis A.H. Sm. were synonymised with *T.citrina* (Smith and Singer 1959). To date, only *T.citrina* and *T.occidentalis* are recognised within the genus *Truncocolumella*.

*Truncocolumellacitrina* is a common ectomycorrhizal species, frequently found in coniferous forests in North America (Zeller 1939, Smith and Singer 1959, Goodman et al. 1998, Wood et al. 1998, Massicotte et al. 2000, Binder and Hibbett 2007, Twieg et al. 2007, Sato and Toju 2019). *Truncocolumellaoccidentalis*, however, is rare and has only been found in Canada (Malençon and Zeller 1940). Until 2012, *Truncocolumella* species had not been reported outside of North America. That year, a specimen was discovered at Mt. Yala in western Sichuan Province, China. Two additional collections were later found in the same region. Western Sichuan Province, part of the Hengduan Mountains, is one of the world's most biodiverse areas. Morphological and molecular analyses revealed that these three samples represent a new species, *T.pseudocolumella*, described in this paper.

## Materials and methods

### Morphological description

The specimens were collected from the Hengduan Mountainous in China. The type and other studied specimens were deposited in the herbarium of Cryptogams of the Kunming Institute of Botany, Academia Sinica (KUN-HKAS).

Descriptions of microscopic and macroscopic characters were based on specimens (HKAS131259 [YAAS L2327], HKAS95533, and HKAS95534) following the methods of Kumar et al. (2017) and Truong et al. (2017). Sections were made with a razor-blade by hand, mounted in a 5% potassium hydroxide (KOH) solution or water and then stained with a cotton blue or lactophenol solution. Mounts were observed under an Olympus BH-2 microscope. Key colours were selected from Kornerup and Wanscher (1978).

### DNA extraction, PCR amplification and sequencing

Total genomic DNA was extracted from the specimens using the OMEGA Plant Genomic DNA Kit. The internal transcribed spacer (ITS) rDNA region was amplified with PCR primers ITS1F and ITS4 (White et al. 1990, Gardes and Bruns 1993, Truong et al. 2017). The large subunit nuclear ribosomal DNA (LSU) region was amplified with the PCR primers LROR and LR5 (Vilgalys and Hester 1990). PCR reactions were performed on a BIO-RAD C1000TM instrument. Thermal cycles with the following settings: initial denaturation for 5 min at 94°C, followed by 32 cycles of 40 s denaturation at 94°C, annealing at 56°C for 40 s for ITS and 52°C for 30 s for LSU, extension for 1 min at 72°C and final extension at 72°C for 10 min. The PCR products were verified on 1% agarose electrophoresis gels stained with ethidium bromide. The purification and sequencing of the PCR products was conducted by Sangon Biotech Limited Company (Shanghai, China).

### Molecular phylogenetic analyses

ITS was used for the analysis of the diversity of *Truncocolumella* species in this study because ITS appears to be a useful locus for the delimitation of *Truncocolumella* species. Thirty ITS sequences from NCBI and this study representing two species of *Truncocolumella* and selected accessions from the closely-related genera *Chroogomphus*, *Gomphidius*, *Rhizopogon*, *Suillus* (Table 1), including *Gastroboletusvividus* Trappe & Castellano, *Gastroboletussubalpinus* Trappe & Thiers and *Gastroboletusruber* (Zeller) Cázares & Trappe as outgroup taxa were used. The sequences of the *Truncocolumella* species generated in this study were submitted to the GenBank database. We first used the Basic Local Alignment Search Tool for the GenBank database to recheck whether the newly-generated sequences were amplified from the contaminant or not and examined clusters with closely-related sequences. DNA sequences were retrieved and assembled using SeqMan. The ITS gene was analysed using BioEdit v. 7 (Hall 2007), sequence alignments were aligned using MAFFT version 7 (Katoh and Standley 2013) and Maximum Likelihood (ML) analysis was performed using RAxML-HPC2 v. 8.2.12 (Stamatakis 2014) as implemented on the CIPRES portal (Miller et al. 2011), with the GTR+G+I model and 1,000 rapid bootstrap (BS) replicates for all genes. A reciprocal 70% bootstrap support approach was used to check for conflicts between the tree topologies from the gene. As the topology of the ML tree and the Bayesian tree are similar, the ITS1, ITS2 and 5.8s sequences were combined using SequenceMatrix (Vaidya et al. 2011) partitioned phylogenetic analyses. For Bayesian Inference (BI), the best substitution model for each partition was determined by MrModelTest 2.2 (Nylander et al. 2004). The result suggested that ITS1: JC+I, 5.8S: GTR+G+I, ITS2: K80+I+G were the best models.. Bayesian analysis was performed using MrBayes ver. 3.2.7a (Ronquist et al. 2011) on the CIPRES Science Portal (Miller et al. 2011) and four parallel runs were performed for 10 million generations sampling every 100^th^ generation for the single gene trees. Parameter convergence > 200 was verified in Tracer v. 1.7 (Rambaut et al. 2018). The phylogenetic clade was strongly supported if the bootstrap support value (BS) was ≥ 70% and/or a posterior probability (PP) < 0.01.

## Taxon treatments

### Truncocolumella pseudocolumella

L. Li, S.H. Li & Y. Wang sp. nov.

0CB67103-CD0B-598F-B525-F13FCBC73E8A

851721

#### Materials

- **Type status:** Holotype. **Occurrence:** catalogNumber: HKAS 131259; recordNumber: L2327; recordedBy: Shu-Hong Li; associatedSequences: KP090063, KP090064; occurrenceID: C1F523CC-76F3-573A-8065-139715C2CC02; **Taxon:** taxonID: MB 851721; scientificNameID: *Truncocolumellapseudocolumella*; **Location:** continent: Asia; country: China; stateProvince: Sichuan; municipality: Ganzi; locality: Yala Snow Mountain; verbatimElevation: 3772.4 m; verbatimLatitude: 30°31′N; verbatimLongitude: 101°37′E; **Identification:** identificationID: HKAS 131259; identifiedBy: Lin Li; **Event:** year: 2012; month: August; day: 19; habitat: in the forest of *Quercusguyavifolia* H. Lév.
- **Type status:** Other material. **Occurrence:** catalogNumber: HKAS 95533; recordedBy: Shan-Ping Wan; associatedSequences: GenBank: OR631922; occurrenceID: E10A3DB1-A334-5221-8BED-5D60FE32F4DA; **Taxon:** scientificName: *Truncocolumellapseudocolumella*; **Location:** higherGeography: Hengduan Mountains area; country: China; stateProvince: Sichuan; county: Ganzi; **Identification:** identificationID: HKAS 95533; identifiedBy: Lin Li; **Event:** year: 2014; month: August; day: 22; habitat: in the forest of *Pinus*
- **Type status:** Other material. **Occurrence:** catalogNumber: HKAS 95534; recordedBy: Shan-Ping Wan; associatedSequences: GenBank: OR631923; occurrenceID: 9ACD3595-0888-58BC-9057-6F0AECF08031; **Taxon:** scientificName: *Truncocolumellapseudocolumella*; **Location:** higherGeography: Hengduan Mountains area; country: China; stateProvince: Sichuan; county: Ganzi; **Identification:** identificationID: HKAS 95534; identifiedBy: Lin Li; **Event:** year: 2014; month: August; day: 22; habitat: in the forest of *Pinus*

#### Description

**Figure 1.**
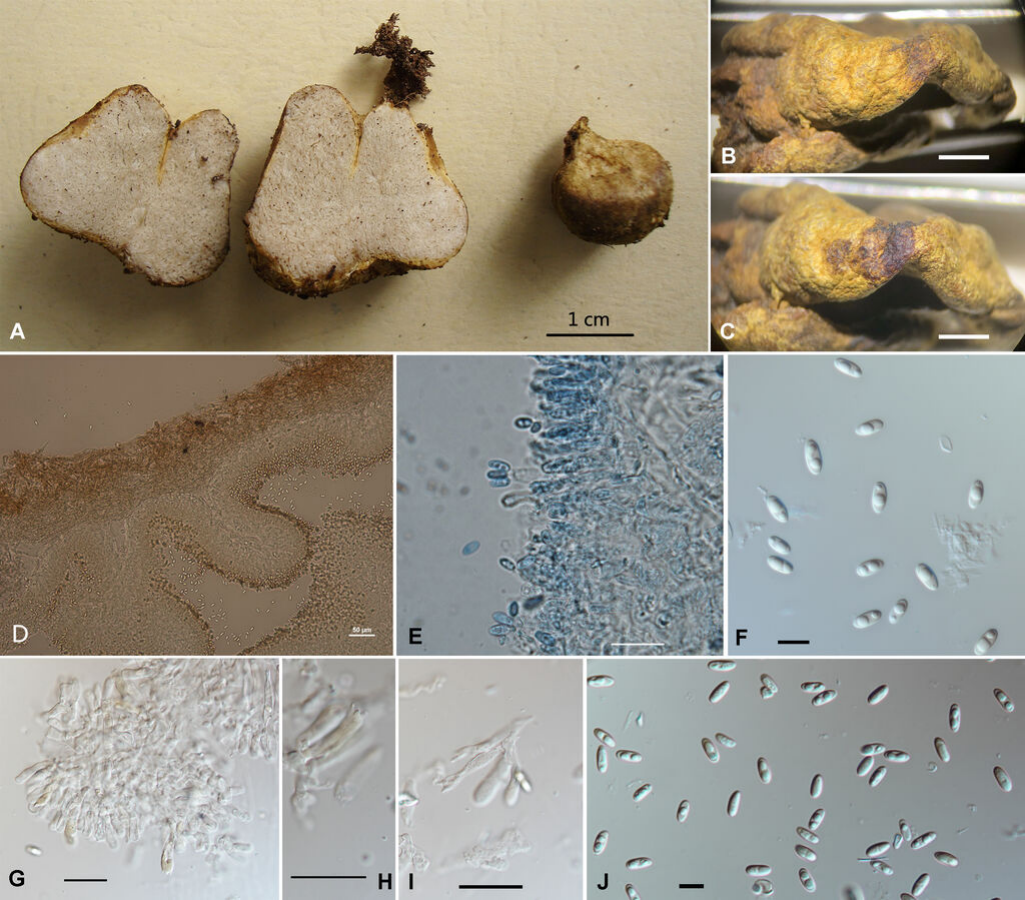
*Truncocolumellapseudocolumella*. **A** Basidiomata; **B, C** Dry peridium turning black when stained with 5% KOH; **D** A section of basidiomata in 5% KOH; **E** A section of the hymenium in lactophenol cotton blue; **G-I** Basidia in 5% KOH (H. 4-spored basidia, I. 2-spored basidia); **F, J** Basidiospores in 5% KOH. Scale bars: A = 1 cm; B, C = 0.5 cm; D = 50 μm; E, G, H, I = 20 μm; F, J = 10 μm.

Basidiomata irregularly depressed-globose to pyriform, with a yellowish-tawny (4C8) rhizomorphic base, 1.5–3.0 cm in diameter, the surface typically smooth and dry in appearance, light yellowish-tawny (4C5) with yellowish-brown (4C8) rhizomorphs, colour unchanged on bruising or exposure, elastic, very mature basidiomata deliquesce like *Rhizopogon* (Fig. 1A). Odour light and pleasant.

Peridium 102–160 μm thick, not separating or evanescent from the gleba at maturity, composed of two layers: outer layer 52–73 μm thick, reddish-tawny (7D7) interwoven hyphae of 1–1.5 μm diameter. The inner layer 40–86 µm thick, consisting of brownish-hyaline nearly parallel interwoven hyphae of 0.5–1.5 μm diameter (Fig. 1D). The boundary between the inner and outer layers gradually transitioning, with the changes in hyphae arrangement direction and hyphae colour. The clamp connection clearly visible, dry peridium becoming black (4F4) when encountering 5% KOH (Fig. 1B and C). Gleba pale white when immature, light brown to tawny at maturity, unchanging on bruising or exposure, cavities relatively small, spongy, lacking obvious columnar or dendroid radiating columella (Fig. 1A). Trama (56.0–) 62.5–85.0 μm, composed of hyaline almost parallel hyphae. Hymenium present on cavities surface. Basidia narrowly clavate, (15.0–) 17.2–19.5 (–22.4) × 3.5–6.5 (–7.0) μm, 2-4-spored. Sterigmata 2–3 μm (Fig. 1E, G, H and I). Basidiospore ellipsoid, smooth, 7.5–10.0 (–11.0) × 4.0–5.0 (–5.6) μm, grey to brownish (7D5) in mass, as seen individually, almost hyaline, typically 1–2 guttulate, infrequently 3-guttulate (Fig. 1F and J), changing to blue in lactophenol cotton blue, not obvious discolouration in Melzer's reagent.

#### Diagnosis

Differs from other species in the genus *Truncocolumella* in the basidiomata devoid of any columnar.

#### Etymology

Pseudocolumella, referring to the absence of the columella.

#### Distribution

China, Sichuan Province.

#### Ecology

Gregarious in the soil associated with *Quercusguyavaefolia* and *Pinus* sp.

#### Notes

Notes: The genus *Truncocolumella* currently includes three species: *T.citrina*, *T.occidentalis* and the newly described *T.pseudocolumella* in this study. Based on the original literature descriptions of the species *T.citrina* (Zeller 1939, Smith and Singer 1959) and the re-examination of the type specimens of *T.citrina* and *T.occidentalis* by Malençon and Zeller (Malençon and Zeller 1940), we compared the key distinguishing features of these three species. In terms of macroscopic characteristics, *T.pseudocolumella* differs from other species in the genus *Truncocolumella* in the basidiomata devoid of any columnar. Furthermore, the basidiomata of *T.citrina* have a diameter of 2–4 cm, which is comparable in size to those of *T.pseudocolumella* (1.5–3 cm), but they are two to three times larger than *T.occidentalis*. The basidiomata surfaces of *T.citrina* are distinctly citrine yellow, those of *T.pseudocolumella* are yellowish-tawny, while *T.occidentalis* has white basidiomata, although the colour of *T.occidentalis* remains uncertain due to preservation in alcohol. Microscopically, *T.citrina* has ellipsoid spores measuring 6–10 × 3.5–5.0 μm, with individual spores appearing nearly hyaline. Similarly, *T.pseudocolumella* has ellipsoid spores measuring 7.5–10.0 × 4.0–5.0 μm, also nearly hyaline, showing close resemblance between the two. According to Malençon's description (Malençon and Zeller 1940), the spores of *T.citrina* are clearly shorter, more oval and of more irregular shape than those of *T.occidentalis*, in which these bodies are long-elliptic or sometimes subcylindrical. Molecular analysis further demonstrates that *T.pseudocolumella* is distinct from *T.citrin*, with high support for their separation into different species.

## Analysis

**Phylogenetic analysis**

**Figure 2.**
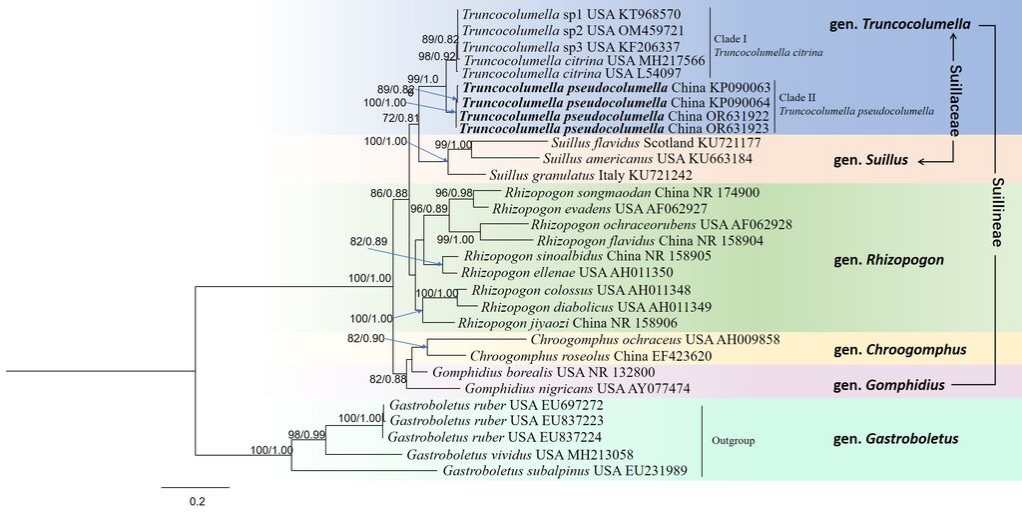
Phylogeny derived from a Maximum Likelihood (ML) analysis of the nrDNA-ITS sequences from Suillineae, including *Truncocolumella* species, using *Gastroboletus* as outgroup. Values next to nodes present Maximum Likelihood bootstrap support values (BS), left and Bayesian posterior probabilities (PP), right. The names of novel species and samples with newly-generated sequences are in bold.

The ML and Bayesian analyses of the 30 ITS sequences are shown in Fig. 2 with associated bootstrap supports for branches.

In the phylogenetic tree, the 25 ITS sequences from Suillineae revealed the phylogenetic relationship of two species of *Truncocolumella* and five *Gastroboletus* sequences are used as outgroups. Since there are few *Truncocolumella* sequences in GenBank and these sequences belong to only one species *T.citrina*, we selected sequences of Suillineae for phylogenetic analysis. The analytics include three sequences of the genus *Suillus*; nine sequences of the genus *Rhizopogon*; two sequences of the genus *Chroogmophus*; two sequences of the genus *Gomphidus*; and nine sequences of the genus *Truncocolumella*. Two clades were revealed in the genus *Truncocolumella*; Clade I includes five sequences of *T.citrina* from the USA; Clade II includes four sequences of a new species, *T.pseudocolumella* from China. The phylogenetic analysis shows that the new species is distinct from *T.citrina*, the type species of *Truncocolumella*. In addition to the ITS sequences used in this phylogenetic analysis, the LSU sequences were amplified from the newly-recorded specimens in this study and uploaded to NCBI for future study.

## Discussion

*Truncocolumella* is an ancient, small genus with only two known species, *T.citrina* and *T.occidentalis*, which were found exclusively on the west Pacific coast of North America until the discovery of the third member, *T.pseudocolumella*, almost a century later in Sichuan, China. *Truncocolumella* is closely related to the epigeous fungi *Suillus* and both genera are currently classified within the family Suillaceae. The genus *Truncocolumella* is characterised by its prominent, stump-dendroid-like columella, a key morphological feature. However, the new species *T.pseudocolumella* has almost no columella, making it similar to species of *Rhizopogon*, although molecular analysis clearly places it within the genus *Truncocolumella*. In addition to the absence of columella in the basidiomata of *T.pseudocolumella*, which is a notable distinguishing feature, *T.citrina* and *T.pseudocolumella* also differ in several morphological characteristics: the basidiomata surface of *T.citrina* is distinctly citrine yellow (Zeller 1939, Smith and Singer 1959), while that of *T.pseudocolumella* is yellowish-tawny. The peridium of *T.citrina* is 70–100 μm thick, whereas that of *T.pseudocolumella* is thicker, reaching 102–160 μm and is differentiated into two layers based on the orientation of hyphae and changes in hyphal colouration. Additionally, the sterigmata of *T.citrina* are 3–5 μm in length, while those of *T.pseudocolumella* are shorter, measuring 1–2 μm in length. Similarly, based on the re-examination of the morphological structures of the type specimens of *T.citrina* and *T.occidentalis* by Malençon and Zeller (Malençon and Zeller 1940) and their descriptions, it is evident that *T.pseudocolumella* has larger basidiomata with a yellowish-tawny surface compared to *T.occidentalis*. Additionally, the spores of *T.occidentalis* are long-elliptic, sometimes almost subcylindrical and are longer than those of both *T.citrina* and *T.pseudocolumella*. *Truncocolumellaoccidentalis* was reclassified from *Dodgeaoccidentalis* and its description is based on a holotype specimen that was preserved in alcohol for 17 years. Since then, no additional reports of this species have been recorded. Unfortunately, molecular data are not available. It is hoped that future collections of this species will help clarify its identity.

*Truncocolumellapseudocolumella* differs significantly from the North American species, which can be attributed to bio-evolutionary geographical isolation and the unique ecological environment of the Hengduan Mountains in China. *Truncocolumellacitrina* is found in coniferous forests of the Pacific Northwest mountains at altitudes around 1200 m (Zeller 1939, Smith and Singer 1959), whereas *T.pseudocolumella* is distributed in alpine *Quercusguyavifolia* and *Pinus* forests at 3700 m in the Hengduan Mountains region of China.

## Supplementary Material

XML Treatment for Truncocolumella pseudocolumella

## Figures and Tables

**Table 1.** Taxa information and GenBank accession numbers of the sequences used in this study. Newly-generated sequences are in bold.

**Species name**	**Voucher**	**Origin**	**GenBank No.**		**Reference**
*Chroogomphusroseolus*	HKAS 50552	China	EF423620	Li et al. (2009)	
*Chroogomphusochraceus*	OKM 25472	USA	AH009858	Miller et al. (2002)	
*Gastroboletusruber*	OSC 79741	USA	EU697272	GenBank	
*Gastroboletusruber*	OSC 69644	USA	EU837224	GenBank	
*Gastroboletusruber*	OSC 74672	USA	EU837223	GenBank	
*Gastroboletussubalpinus*	Trappe607-holotype	USA	EU231989	Dentinger et al. (2010)	
*Gastroboletusvividus*	JLF4456	USA	MH213058	GenBank	
*Gomphidiusborealis*	IB:NR19990532-holotype	USA	NR_132800	Miller et al. (2002)	
*Gomphidiusnigricans*	OKM 27830	USA	AY077474	Miller et al. (2002)	
*Rhizopogoncolossus*	MICH AHS49480-holotype	USA	AH011348	Grubisha et al. (2002)	
*Rhizopogondiabolicus*	MICH AHS68424-paratype	USA	AH011349	Grubisha et al. (2002)	
*Rhizopogonellenae*	MICH AHS66137-holotype	USA	AH011350	Grubisha et al. (2002)	
*Rhizopogonevadens*	MICH AHS65484-holotype	USA	AF062927	Grubisha et al. (2002)	
*Rhizopogonflavidus*	YAAS L2957	China	NR_158904	Li et al. (2016)	
*Rhizopogonjiyaozi*	YAAS L2929	China	NR_158906	Li et al. (2016)	
*Rhizopogonochraceorubens*	MICH AHS59643-holotype	USA	AF062928	Grubisha et al. (2002)	
*Rhizopogonsinoalbidus*	YAAS L2949	China	NR_158905	Li et al. (2016)	
*Rhizopogonsongmaodan*	HKAS 106767	China	NR_174900	Wang et al. (2021)	
*Suillusamericanus*	C0075059F	USA	KU663184	Zhang et al. (2022)	
*Suillusflavidus*	KM171907	Scotland	KU721177	Zhang et al. (2022)	
*Suillusgranulatus*	KM172141	Italy	KU721242	Zhang et al. (2022)	
*Truncocolumellacitrina*	JLF 2149	USA	MH217566	GenBank	
*Truncocolumellacitrina*	TDB-2001	USA	L54097	Kretzer et al. (1996)	
***Truncocolumellapseudocolumella***	**HKAS131259**	**China**	**KP090063**	**This study**	
***Truncocolumellapseudocolumella***	**HKAS131259**	**China**	**KP090064**	**This study**	
***Truncocolumellapseudocolumella***	**HKAS95533**	**China**	**OR631922**	**This study**	
***Truncocolumellapseudocolumella***	**HKAS95534**	**China**	**OR631923**	**This study**	
*Truncocolumella* sp1.	OSC 67369	USA	KT968570	GenBank	
*Truncocolumella* sp2.	MR3D88-RHITRU1	USA	OM459721	GenBank	
*Truncocolumella* sp3.	OSC 111948	USA	KF206337	GenBank	
